# Rinsing a Pandemic Down: Effects of Oral Hygiene in SARS-CoV-2: A Two-Center Prospective Pilot Study

**DOI:** 10.3390/jcm14238280

**Published:** 2025-11-21

**Authors:** Philipp Ehrmann, Carolin Goetz, Holger Bock, Lena Denk, Petr Posta, Herbert Deppe, Elisabeth Maier, Oliver Bissinger

**Affiliations:** 1 Department of Oral and Maxillofacial Surgery, Medical University Innsbruck, Anichstraße 35, 6020 Innsbruck, Austria; 2 Department of Oral and Maxillofacial Surgery, TUM School of Medicine and Health, Technical University of Munich, Klinikum Rechts der Isar, Ismaninger Strasse 22, 81675 Munich, Germany; 3 Office for Knowledge and Technology Transfer, University of Innsbruck, Technikerstr. 21a, 6020 Innsbruck, Austria; 4 Department of Stomatology, University Hospital Pilsen, Faculty of Medicine, Charles University, 32300 Pilsen, Czech Republic

**Keywords:** COVID-19, dental infection control, mouth rinse, oral hygiene, saliva testing, SARS-CoV-2

## Abstract

**Background:** Saliva sampling is increasingly used for respiratory virus diagnostics in dentistry and oral medicine due to patient comfort and reduced exposure risk. How routine behaviors—mechanical oral hygiene, rinsing, and food intake—affect short-term SARS-CoV-2 detectability remains insufficiently characterized for clinical workflows. **Methods:** In this international two-center pilot study, twelve RT-PCR-confirmed COVID-19 patients provided paired mouth-rinse saliva samples and pharyngeal swabs at predefined time points. The study assessed (I) an intensified 3 min mechanical oral hygiene protocol (toothbrushing of teeth, gingiva, tongue, and palate, followed by toothpaste–saliva gargling); (II) repeated short mouth rinses; and (III) postprandial sampling. Viral RNA was quantified by RT-PCR; Ct-trajectories were analyzed intra-individually. **Results:** Cycle threshold (Ct) values from pharyngeal swabs remained relatively stable over time, whereas mouth-rinse samples exhibited notable fluctuations throughout the 24 h period. An average increase of 3 Ct units was observed three minutes after the final mouth rinse (T24+3). Meal ingestion was associated with increased Ct values, rising by 4–5 units for pharyngeal swabs and 3–11 units for mouth rinses immediately after eating. **Conclusions:** In clinical dental settings, saliva diagnostics are feasible but acutely modulated by common behaviors. Mechanical oral hygiene, brief rinsing, and food intake can transiently reduce detectable oral SARS-CoV-2 RNA, with potential implications for timing of sampling, chairside triage, and infection-control protocols. This pilot study provides initial evidence supporting the development of standardized pre-analytical instructions (e.g., fasting window, pre-rinse policy, and sampling timing relative to oral hygiene and meals) to enhance the reliability of saliva-based testing in dental care.

## 1. Introduction

Since the spring of 2003, coronaviruses have emerged as significant human pathogens, beginning with the Severe Acute Respiratory Syndrome (SARS) outbreak caused by the novel SARS-CoV [1]. This highlighted coronaviruses’ potential to cause severe disease in humans [2]. In April 2012, a second zoonotic coronavirus—Middle East Respiratory Syndrome Coronavirus (MERS-CoV)—was identified. Primarily transmitted from dromedary camels to humans, MERS-CoV caused severe respiratory infections, with a case fatality rate of approximately 35–37% [3]. The global relevance of coronaviruses reached unprecedented levels in December 2019 with the emergence of Coronavirus Disease 2019 (COVID-19), caused by SARS-CoV-2, in Wuhan, China. The ensuing pandemic profoundly affected public health, healthcare systems, economies, and societies worldwide. By October 2020, more than 36.5 million individuals had been infected, with over one million deaths reported globally [4]. Healthcare infrastructures were overwhelmed, medical resources strained, and essential services disrupted. In response, mitigation strategies, including large-scale vaccination campaigns, social distancing mandates, and reinforcement of healthcare capacities, were implemented [5].

SARS-CoV-2 is predominantly transmitted via respiratory droplets during speaking, breathing, coughing, or sneezing. Indirect transmission through contaminated surfaces has also been documented, prompting stringent hygiene protocols [6,7].

A thorough understanding of SARS-CoV-2 transmission dynamics is essential to prevention. Effective measures include social distancing, rigorous hand hygiene, surface disinfection, and consistent use of personal protective equipment [8]. Nosocomial transmission remains a major risk, as viral RNA has been detected on hospital surfaces—such as toilets and patient rooms—and in air samples, highlighting the necessity for strict infection control [9]. To mitigate future outbreaks, it is essential to recognize transmission routes early and strengthen hospital hygiene practices [10]. Timely and precise identification of infected individuals remains challenging. False-negative nasopharyngeal or sputum tests have led to premature discharges and previously undetected transmission, underscoring the need for repeated and comprehensive diagnostic strategies [2].

Reverse-transcriptase polymerase chain reaction (RT-PCR) remains the diagnostic gold standard for COVID-19, typically performed on naso- or oropharyngeal swabs [11,12,13]. Whole-genome sequencing enhances identification specificity via targeted amplification of viral genes. Additionally, SARS-CoV-2 RNA can be reliably detected in specimens such as saliva, lower respiratory tract secretions, stool, urine, serum, and plasma, offering diagnostic sampling flexibility [4].

In 2018, To et al. evaluated the utility of saliva as a diagnostic specimen for respiratory viruses using an automated multiplex molecular assay, comparing results from saliva and nasopharyngeal aspirate (NPA) samples [14]. The study revealed a 93.3% concordance between saliva and NPA in detecting respiratory pathogens. During the COVID-19 pandemic, the same group extended their research to SARS-CoV-2, reporting over 90% agreement between the two sample types. Notably, all 33 patients with negative NPA results also tested negative using saliva; however, some individuals tested positive in saliva only, suggesting potential advantages in diagnostic sensitivity. Saliva specimens underwent total nucleic acid extraction using lysis buffer, followed by reverse transcription polymerase chain reaction (RT-PCR) for viral load quantification.

Saliva sampling offers several practical benefits over nasopharyngeal swabbing (NPS). It is less invasive, more comfortable for patients, and allows self-collection with minimal instruction. Furthermore, it reduces the need for trained personnel and lowers the risk of nosocomial exposure, thus enhancing overall testing efficiency [15].

Maintaining adequate oral hygiene is considered critical in reducing both infection risk and viral transmission via the oral cavity. In a study by Kawamoto et al., poor oral hygiene was associated with significantly increased susceptibility to influenza infection—an effect comparable to age as a risk factor. Conversely, elevated levels of salivary proteins and weakly acidic saliva were associated with decreased infection risk [16]. These findings emphasize the potential relevance of oral health in viral disease prevention.

Given the role of the oral cavity as a primary entry and replication site for respiratory viruses [17], it is clinically pertinent to investigate whether mechanical oral hygiene measures, such as tooth brushing and over-the-counter mouth rinses, can reduce SARS-CoV-2 viral load. Improved oral hygiene may not only reduce false-negative test results but also lower transmission risk. While individual components of certain mouth rinses have shown efficacy in decreasing viral load, data on the effect of tooth brushing remain limited [18,19].

This study aimed to evaluate whether dental hygiene influences intraoral viral load. The null hypothesis stated that oral cleaning does not affect the cycle threshold (Ct) values obtained from COVID-19 PCR assays.

## 2. Materials and Methods

### 2.1. Ethics

The study was approved by the local ethics committees of Munich (Ethics Approval No. 33/20 S, 15 June 2020) and Innsbruck (Ethics Approval No. 1231/2020, 14 September 2020). It was conducted in full accordance with the principles of the Declaration of Helsinki. Written informed consent was obtained from all participants prior to inclusion in the study.

### 2.2. Study Design and Subjects

This prospective, two-center pilot study investigates the short-term effect of mechanical oral cleaning on the SARS-CoV-2 viral load in the oral cavity and oropharynx. The primary objective is to determine whether a standardized three-minute brushing protocol using a commercially available toothbrush and toothpaste leads to a measurable reduction in viral concentration. Viral loads are assessed via quantitative RT-PCR from paired throat swabs and mouth rinse samples collected at predefined time points before and after the intervention. As a secondary objective, the study explores the detectability of SARS-CoV-2 RNA from both sample types over time, aiming to provide insights into viral load dynamics in relation to oral hygiene practices.

Twelve hospitalized adult patients with laboratory-confirmed SARS-CoV-2 infection were enrolled between 2021 and 2022 at the Medical University of Innsbruck (Austria) and the Klinikum rechts der Isar, Technical University of Munich (Germany). The study was conducted as a two-center pilot investigation with an exploratory design; therefore, no formal power calculation was carried out. Inclusion criteria comprised age ≥18 years and a positive RT-PCR result from a nasopharyngeal swab. Exclusion criteria included cognitive impairment, inability to cooperate, or the requirement for mechanical ventilation. Demographic and clinical data, including sex, age, and relevant laboratory parameters, were obtained from electronic medical records. All personal identifiers were removed and replaced with pseudonymized codes to ensure confidentiality. Data analysis was performed using exclusively pseudonymized datasets.

To investigate the effects of different oral interventions, participants were allocated to one of three subgroups according to their clinical setting and feasibility, rather than through formal randomization. This approach was chosen due to the strict infection control measures and logistical constraints during the COVID-19 pandemic, which made randomized allocation unfeasible. The first group (*n* = 6) performed thorough mechanical oral hygiene followed by gargling with a mixture of toothpaste, saliva, and water. The second group (*n* = 4) underwent oral cleaning through meal ingestion, with sampling conducted before and at multiple time points following food intake. As all participants were hospitalized, standardized meals with identical nutritional composition and timing were provided to ensure comparable metabolic conditions. The third group (*n* = 2) rinsed the oral cavity with 10 mL of water, including intermittent gargling over short intervals. The interventions aimed to reflect real-life behaviors that may influence the oral viral environment.

Intraindividual comparisons of cycle threshold (Ct) values before and after the respective interventions were used to evaluate the potential of these measures to transiently lower viral loads. This study provides exploratory evidence on the role of oral hygiene and behavioral factors in modulating SARS-CoV-2 presence in the oral cavity. The findings may contribute to the development of adjunctive clinical recommendations aimed at minimizing viral transmission risk and enhancing the diagnostic reliability of oral-based sampling methods.

### 2.3. Intensified Oral Hygiene Protocol

Participants received a soft-bristled toothbrush (elmex^®^ SENSITIVE, GABA International AG, Therwil, Switzerland) and fluoridated toothpaste (elmex^®^ KARIESSCHUTZ, GABA International AG, Therwil, Switzerland), along with standardized written and verbal instructions for performing oral hygiene.

The cleaning procedure followed a fixed sequence targeting each quadrant of the oral cavity—upper right, upper left, lower left, and lower right—in a defined order. Participants were instructed to thoroughly clean the occlusal, buccal, and lingual surfaces of the teeth, as well as the gingival margins. The protocol further included brushing of the tongue, performed from posterior to anterior, and gentle cleaning of the hard palate to enhance overall microbial reduction.

All individuals were instructed to continue brushing for a total duration of three minutes. To ensure consistency across participants, identical toothbrushes and toothpaste were used, and the oral hygiene routine was standardized. The amount of toothpaste could vary slightly between the participants. Following the brushing procedure, participants gargled a mixture of remaining toothpaste, saliva, and a sip of water three times for approximately three seconds each. This was followed by a prolonged rinse with the same mixture for 30 s. Subsequently, participants were instructed to spit out the mixture and rinse their mouths with clean water three times to remove residual substances. The comprehensive and standardized nature of this protocol aimed to maximize oral cleanliness and ensure reliable assessment of its potential effect on SARS-CoV-2 viral load.

### 2.4. Specimen Collection and Sampling Protocols

Patients were eligible for inclusion if they had a confirmed SARS-CoV-2 infection, verified by RT-PCR testing of nasopharyngeal swabs. The study comprised three subgroups, each with a distinct sampling scheme to assess the short-term effects of oral interventions on SARS-CoV-2 viral concentration in the oral cavity.

In the first subgroup, participants followed the intensified oral hygiene protocol. Saliva specimens were collected at multiple defined time points. A baseline sample (T–1) was obtained in the morning prior to any oral hygiene activities. Immediately after completion of the cleaning protocol, a second sample was taken (T0), followed by additional samples at 4, 8, 12, and 24 h post-intervention (T4, T8, T12, T24). An additional sample was collected at 24 h and 3 min (T24+3) to verify consistency.

The second subgroup was designed to evaluate the effect of food ingestion and rinsing behavior on viral load. Mouth-rinse samples were collected at six defined time points (A through F), beginning at approximately 10 a.m., roughly two hours after breakfast. Participants in this group used 10 mL of water for rinsing. Participants 7 and 8 rinsed for 10 s, while participants 9 and 10 rinsed for 30 s. Notably, participant 10 had an extended interval of 44 min between rinse points D and E, potentially introducing variability. To evaluate the effect of food intake, samples were also obtained before the meal (M–1), immediately after eating (M0), and at 30, 60, 90, and 120 min post-meal (M30, M60, M90, M120).

In the third subgroup, participants completed the mouth-rinse procedure and subsequently rinsed their mouth with two sips of water to remove any remaining residues, ensuring sample clarity and standardization.

### 2.5. Saliva Collection and Processing Protocol

To ensure consistent sample acquisition, participants were instructed to rinse the oral cavity with 10 mL of water for one minute before expectorating into a sterile collection container. A 0.5 mL aliquot of the collected saliva was subsequently transferred into an Eppendorf tube containing 0.5 mL of virus-inactivating buffer. This standardized pre-analytical procedure enabled reproducible sample handling across all participants.

### 2.6. Laboratory Analysis

All specimens were transferred directly to the diagnostic laboratory and processed within two hours of collection using a validated testing system (PurFlock Ultra^®^ swab, Puritan Medical Products, Guilford, ME, USA; 1.2 mL virus transport medium, Puritan Medical Products, Guilford, ME, USA). During interim handling, samples were kept refrigerated at 4 °C to maintain RNA stability. Both throat swabs and saliva samples were subjected to molecular and virological analysis for SARS-CoV-2 detection and infectivity assessment.

For viral RNA quantification, the RealStar^®^ SARS-CoV-2 RT-PCR Kit (Altona Diagnostics GmbH, Hamburg, Germany) was employed.

Total RNA was extracted from swab and saliva samples using the IndiSpin Pathogen Kit (Indical Bioscience GmbH, Leipzig, Germany)under sterile clean-room conditions. An internal positive control was added to each sample during extraction to monitor for inhibition or loss of sensitivity. Extracted RNA was then analyzed by RT-PCR using the same platform and reagent lots to ensure inter-run comparability. Appropriate positive and negative controls were included to ensure the validity and reproducibility of all PCR results.

### 2.7. Statistical Analysis

All statistical analyses were performed using Microsoft Excel (Version 16.11.1, Microsoft Corporation, Redmond, WA, USA) and IBM SPSS Statistics (Version 25, IBM Corporation, Armonk, NY, USA). For continuous variables, group means and standard deviations were calculated across all three intervention groups. Time-series data for each individual enabled estimation of intraindividual Ct value dynamics and the potential average treatment effect of intensified oral hygiene. Categorical variables were analyzed by calculating absolute frequencies.

The dataset was initially tested for normal distribution using the Shapiro–Wilk test. For variables not normally distributed, non-parametric methods were applied. Comparisons between two independent groups were conducted using the Mann–Whitney U test. A *p*-value of less than 0.05 was considered statistically significant. To visualize treatment effects over time, line graphs were generated, illustrating Ct value trajectories in relation to the timing of each intervention.

## 3. Results

### 3.1. Combined Group Analysis

The study included twelve hospitalized patients with RT-PCR–confirmed SARS-CoV-2 infection. A total of 34 paired samples were obtained, each consisting of a standardized 10 s mouth rinse followed immediately by a pharyngeal swab. Viral RNA concentrations were determined using reverse transcription polymerase chain reaction (RT-PCR), with results expressed as cycle threshold (Ct) values. Because Ct values are inversely proportional to viral load, lower Ct values correspond to higher concentrations of viral RNA. In cases where SARS-CoV-2 RNA was undetectable, a Ct value of 44 was assigned to ensure consistency in statistical comparisons.

**Figure 1:**
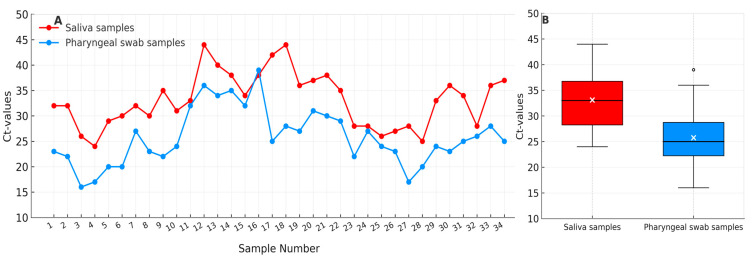
Comparison of cycle threshold (Ct) values from paired saliva (red) and pharyngeal swab (blue) samples (*n* = 34). (**A**) Individual paired Ct values illustrating consistently higher Ct values in saliva compared with corresponding pharyngeal swabs. (**B**) Boxplot summary showing median (black line), mean (white ×), interquartile range (IQR), and 1.5 × IQR whiskers. Saliva samples demonstrate systematically higher Ct values (mean 33.1 ± 5.3) than pharyngeal swabs (mean 25.8 ± 5.5), indicating lower viral RNA concentrations in saliva (*p* < 0.05).

Saliva samples showed systematically higher Ct values (mean ± SD = 33.1 ± 5.3) than corresponding pharyngeal swabs (25.8 ± 5.5), indicating lower viral RNA concentrations in saliva. The observed difference was statistically significant (Mann–Whitney U = 964.0, *p* < 0.001) with a large effect size (r = 0.57). The 95% confidence intervals for the mean Ct values were 31.3–35.0 for saliva and 23.8–27.7 for pharyngeal swabs. These findings are consistent with Figure 1, which illustrates the systematic difference in Ct value distribution between both sampling methods.

### 3.2. Subgroup Analysis: Intensified Oral Hygiene (n = 6)

Participants in this subgroup underwent standardized mouth-rinse and pharyngeal swab sampling at seven defined time points: before oral hygiene (T–1), immediately after the intervention (T0), and subsequently at 4, 8, 12, and 24 h, as well as 24 h and 3 min post-intervention (T24+3).

**Figure 2:**
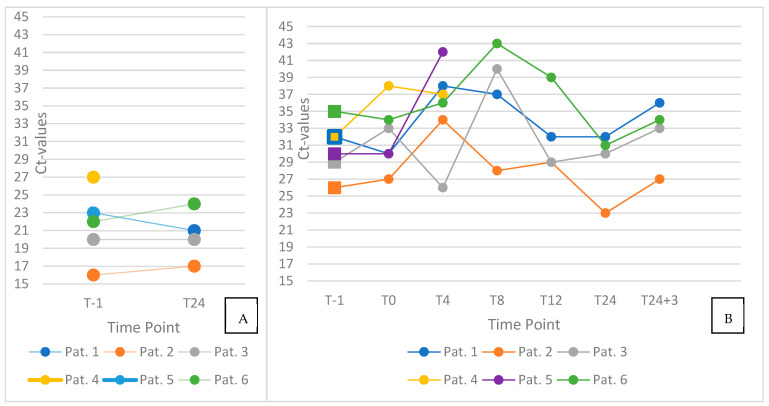
Individual trajectories of cycle threshold (Ct) values in pharyngeal swab (left) and saliva (right) samples. Panel (**A**) displays paired pharyngeal swab results obtained before oral hygiene (T–1) and at 24 h post-intervention (T24). Panel (**B**) illustrates longitudinal Ct values from saliva samples (*n* = 6) collected at seven standardized time points: before oral hygiene (T–1), immediately after the intervention (T0), and at 4, 8, 12, and 24 h, as well as 24 h + 3 min post-intervention (T24 + 3). Ct values of pharyngeal swabs remained largely stable, whereas saliva samples displayed greater temporal variability throughout the observation period.

Pharyngeal swab Ct values remained relatively stable throughout the 24 h observation period (Figure 2, left panel), showing only minor intraindividual variation. In contrast, mouth-rinse samples demonstrated greater temporal fluctuation (Figure 2, right panel), with Ct values varying more noticeably across time points.

When comparing pre- and immediate post-intervention values (T–1 vs. T0), an increase in Ct values—suggestive of a short-term reduction in viral load—was observed in three of six participants, with Ct elevations of 1, 4, and 6 units, respectively. In the remaining three individuals, no relevant change or slight decrease in Ct values was recorded following the intervention.

At later time point measurements in four patients, after two patients did not want to participate anymore, particularly at T8 (which coincided with lunch), two participants showed peaking Ct values in mouth-rinse samples. Additionally, Ct values at T24+3 were consistently higher than those at T24 in all participants, with an average increase of approximately 3 units.

### 3.3. Multicenter Comparison: Munich Cohort (n = 4)

To validate the findings from the Innsbruck cohort, additional data were collected from a second center at the Klinikum rechts der Isar, Technical University of Munich. Four patients with RT-PCR-confirmed SARS-CoV-2 infection via nasopharyngeal swab underwent the same standardized oral hygiene protocol, consisting of mechanical cleaning of the teeth, gingiva, tongue, and palate, followed by gargling with a toothpaste–saliva mixture. Saliva samples were collected immediately before (T–1) and after (T0) the intervention.

At baseline (T–1), all four patients tested positive for SARS-CoV-2 RNA in saliva. Following the oral hygiene procedure (T0), none of the samples showed detectable viral RNA, resulting in a complete loss of detectability within the sensitivity limits of the assay. Outcomes were documented in a binary format (positive or negative). For comparative analysis, a Ct value < 30 was defined as positive.

**Figure 3:**
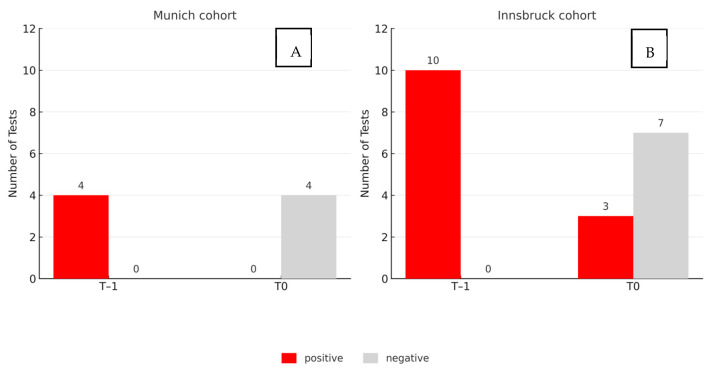
Results before (T–1) and after (T0) standardized oral hygiene in two independent cohorts. (**A**): Munich (*n* = 4); (**B**): Innsbruck (*n* = 10). Bars indicate saliva samples testing positive (red) or negative (gray) for SARS-CoV-2 RNA (Ct < 30 considered positive). Viral detectability decreased in 70% of samples in Innsbruck and was completely lost in Munich.

In the Innsbruck cohort, 10 participants presented with positive saliva samples at T–1. Of these, seven tested negative at T0, corresponding to a reduction in viral detectability in 70% of the cases. The main results are summarized in Figure 3.

### 3.4. Subgroup Analysis: Meal and Mouth-Rinsing (n = 4)

**Figure 4:**
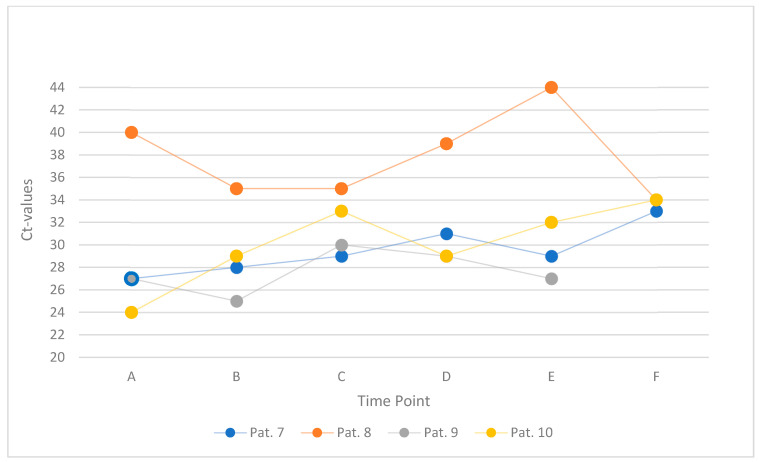
Ct values within one hour, starting two hours after breakfast, in four individual patients (Patients 7–10). Each line represents one participant’s serial mouth-rinse measurements (six time points, A–F).

This subgroup comprised four patients (Patients 7 to 10) who underwent serial mouth-rinse testing at six defined time points (labeled A to F), beginning approximately two hours after breakfast. Patients 7 and 8 performed a 10 s rinse with 10 mL of water, whereas Patients 9 and 10 rinsed for 30 s using the same volume. The standard interval between samples was three minutes. A slight deviation from this protocol occurred in Patient 10, who exhibited a prolonged interval of 44 min between rinses D and E. Figure 4 offers a graphical summary of the study’s findings.

A total of 22 Ct values were recorded across all patients and time points. Five of six values were available for Patient 9 due to missing data at time point F. In 11 of these, an increase in Ct value was observed relative to the preceding sample, with changes ranging from 1 to 5 units. In the remaining samples, Ct values either remained unchanged or decreased by up to 5 units (one outlier with 10 units).

### 3.5. Subgroup Analysis: Postprandial Viral Load Dynamics (n = 3)

**Figure 5:**
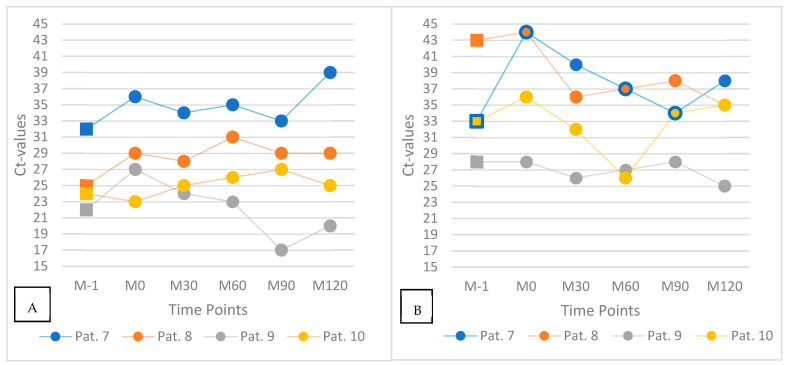
Ct values after ingestion of a meal: pharyngeal swab (**A**) and saliva mouth-rinse samples (**B**). Each line represents one individual patient (Patients 7–10) measured at six standardized time points: before the meal (M–1), immediately after ingestion (M0), and 30, 60, 90, and 120 min postprandially (M30–M120). Squares denote pre-meal samples; dots represent postprandial time points.

To evaluate the potential influence of food intake on SARS-CoV-2 viral load in the oral cavity and oropharynx, four patients (Patients 7 to 10) underwent both pharyngeal swab and mouth-rinse sampling at six standardized time points relative to lunch: immediately before the meal (M–1), directly after ingestion (M0), and at 30, 60, 90, and 120 min postprandially (M30 to M120). The temporal development of Ct values is depicted in Figure 5.

At time point M0, three of the four patients (Patients 7, 8, and 9) showed marked increases in Ct values compared to M–1. In pharyngeal swabs, Ct values rose by approximately 4 to 5 units, and in mouth-rinse samples, increases ranged from 3 to 11 units. In contrast, Patient 10 exhibited a slight decrease of 2 Ct units immediately after food intake. In the following time points, Ct values gradually declined in most participants, approaching pre-meal levels by M120 in several cases. Patient 10, who initially showed a lower Ct at M0, exhibited a delayed increase over the subsequent time course.

While the general trend of postprandial Ct elevation followed by a gradual return toward baseline was observed in most patients, considerable inter- and intraindividual variation in viral kinetics was noted. This variability may be influenced by individual differences in salivary flow, viral shedding dynamics, meal composition, or sample handling procedures.

### 3.6. Subgroup Analysis: Mouth-Rinsing with Gargling (n = 2)

To explore the potential impact of pre-rinse procedures on the consistency of saliva-based SARS-CoV-2 detection, two participants (Patients 11 and 12) performed a standardized water-based mouth rinse with gargling prior to repeated sampling. Both individuals rinsed and gargled with 10 mL of water for 50 s before each sampling time point. Samples were collected at intervals A to D, followed by a 90 min pause, and resumed at time points E to H with continued 3 min intervals. Prior to the first sample, both patients also rinsed with two sips of water to eliminate residual mucus or virus-laden secretions that could affect specimen quality or Ct value interpretation.

**Figure 6:**
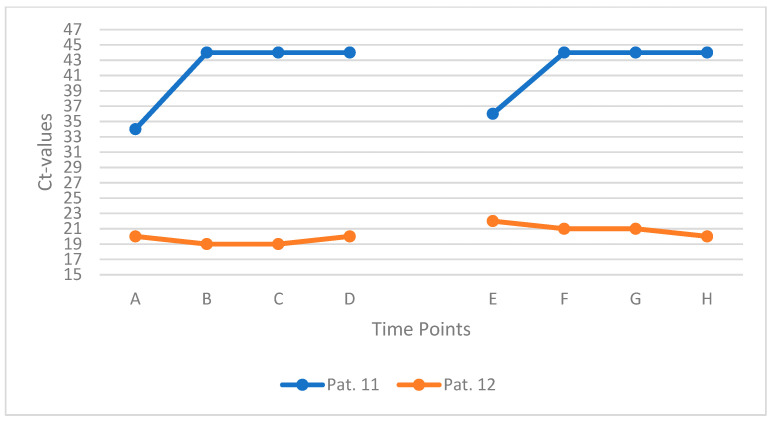
Ct values of two patients gargling with water before saliva sampling. Each line represents one individual patient (Patients 11 and 12) measured at eight consecutive time points (A–H).

Patient 11, who presented with a high initial Ct value consistent with low viral RNA concentration, showed no detectable SARS-CoV-2 RNA in subsequent samples after the first rinse and gargle. In contrast, Patient 12 had a low initial Ct value and exhibited no meaningful change over the full observation period. Across all time points, intraindividual Ct variation remained within a narrow range of ±2 units for both patients. Figure 6 provides an overview of the key findings.

## 4. Discussion

This multicenter observational study investigated the temporal dynamics and detectability of SARS-CoV-2 RNA in saliva using standardized mouth-rinse sampling protocols. The consistently lower Ct values observed in oral rinse specimens compared to pharyngeal swabs across the full cohort suggest a higher detectable viral load in the oral cavity at the time of sampling. These findings align with previous research emphasizing the sensitivity of saliva-based testing—particularly during early or active phases of infection—and support the use of mouth-rinse saliva samples as a feasible alternative to reduce nosocomial transmission risk [12]. In our cohort, saliva and pharyngeal swab testing showed >90% concordance, further supporting the clinical applicability of this non-invasive method. Given its improved patient comfort, potential for self-collection, and reduced exposure risk for healthcare workers, mouth-rinse sampling may be especially valuable in mass-screening or outpatient settings [12,20]. However, challenges remain. Saliva-based diagnostics are subject to procedural and physiological influences that can affect sample quality. These include potential dilution of viral RNA due to rinsing, variability in hydration status, recent food intake, and fluctuations in salivary flow [21]. Standardized collection methods—such as fasting periods prior to sampling or the use of optimized collection tools—may help address these limitations and improve reproducibility in clinical and research contexts. As this study was designed as an exploratory pilot with a limited number of paired samples (*n* = 34), the calculated effect size and confidence intervals should be interpreted cautiously. These statistical estimates primarily serve to complement the graphical representation of variability (Figure 1) and to provide an approximate quantitative indication of the observed differences rather than inferential evidence. Given the small sample size, non-parametric tests and variability visualization were prioritized over formal parametric inference. This approach aligns with the exploratory intent of the study, which is emphasized throughout the manuscript.

The ability of SARS-CoV-2 to be transmitted by asymptomatic individuals—unlike the virus responsible for the 2002/2003 SARS outbreak—further underscores the importance of early detection strategies [22]. Non-invasive methods such as saliva testing could play a critical role in identifying infectious individuals in pre- or asymptomatic stages and limiting viral spread [23]. Our data further indicate that mechanical oral hygiene may transiently reduce detectable viral load in the oral cavity, as shown in the literature [24,25].

Data from the intensified oral hygiene subgroup revealed a heterogeneous response: only three of six patients exhibited a relevant Ct increase post-intervention, indicating a short-term reduction in viral load. However, fluctuations in Ct values were observed at later time points—particularly at T8, which coincided with food intake—and again consistently at T24+3, three minutes after the final time point. These short-term changes suggest that behavioral factors such as mastication, swallowing, and fluid interaction can transiently alter viral detectability, likely by mechanically displacing virus-containing secretions or enhancing salivary flow. Thus, PCR tests should not be performed immediately after eating.

This interpretation is supported by the postprandial subgroup analysis, where three of four patients showed increased Ct values immediately after eating. The observed transient reduction in detectable viral RNA likely results from stimulated salivary flow, elevated lysozyme activity, and pH-related changes that may reduce viral viability [26]. In most patients, Ct values returned toward baseline within 90 to 120 min, consistent with reaccumulation of viral particles or renewed mucosal shedding. Nevertheless, the interindividual variation observed highlights the importance of controlling for the timing of last food intake when collecting saliva-based diagnostic samples.

A separate subgroup that underwent repeated rinsing and gargling demonstrated minimal intraindividual Ct variation across time points. Although the overall impact on viral load was limited—likely depending on initial viral burden—rinsing appeared to enhance sample consistency. One patient’s Ct value dropped below the assay’s sensitivity threshold post-rinse, while another maintained a high viral load despite repeated rinsing. These findings suggest that preparatory rinsing protocols, although not markedly reducing viral RNA concentration, may still improve the reliability of test results and should be considered in future diagnostic workflows.

Interestingly, Ct values from saliva were generally higher than those from pharyngeal swabs in our cohort, likely reflecting dilution due to the standardized 10 mL mouth-rinse protocol. Only one patient (Patient 7) exhibited the inverse pattern. This observation highlights the importance of accounting for procedural dilution when interpreting saliva-based viral load data.

Taken together, our findings demonstrate that saliva-based SARS-CoV-2 detection is feasible and potentially more sensitive than pharyngeal swabs. However, its diagnostic reliability depends heavily on pre-analytical conditions, including food intake, hydration, rinsing behavior, and oral hygiene practices. Accordingly, we recommend that saliva samples should not be collected immediately after food intake or oral hygiene procedures. Saliva sampling, in general, should be more standardized to receive valid results. As SARS-CoV-2 transitions into endemicity, these insights remain relevant for optimizing saliva-based diagnostics—not only for COVID-19 but for future respiratory pathogens that may follow a similar transmission profile. Studying interventions that reduce viral load—such as those explored here—can improve diagnostic accuracy and help mitigate transmission risk. These findings support integrating oral hygiene and standardized collection protocols into broader infection control strategies in preparation for future pandemics.

### Limitations

This study has several limitations that warrant consideration. First and foremost, the small sample size of this pilot study limited the statistical power and precluded the use of inferential analyses beyond descriptive trends. As a pilot study, the primary objective was exploratory—to assess feasibility and generate hypotheses for future research—rather than to establish definitive causal relationships.

Patient recruitment was significantly hampered by the evolving trajectory of the COVID-19 pandemic. Increasing population-level immunity, primarily through vaccination, and the emergence of the less virulent Omicron variant reduced both clinical severity and public concern around transmission. These factors led to a sharp decline in hospitalization rates, thereby narrowing the available pool of eligible participants. As a result, data collection was concluded in September 2022, curtailing the opportunity to expand the cohort and stratify by clinical severity, viral variant, or vaccination status.

Methodologically, the study relied on RT-PCR testing of mouth-rinse and pharyngeal swab samples, with Ct values used as surrogate markers for viral load. However, Ct values are influenced by several pre-analytical and analytical variables, including sample viscosity, pipetting accuracy, and instrument calibration. The use of a fixed Ct cutoff (e.g., 44 for non-detectable samples) may also introduce artificial ceiling effects and reduce sensitivity to small but biologically relevant differences.

Moreover, intraindividual variability in sampling—such as differences in rinse intensity, swallowing between rinses, or adherence to fasting instructions—may have affected sample consistency. Despite protocol standardization, behavioral factors (e.g., food intake, oral habits) likely introduced noise into the data and limited reproducibility. The findings from behavioral subgroups, while suggestive, should therefore be interpreted with caution due to their exploratory nature and limited generalizability.

Finally, the study population consisted exclusively of hospitalized patients, which may not reflect the broader ambulatory or asymptomatic population. Generalization of the results to community-based screening or surveillance settings is therefore limited. Future studies should aim to include more diverse cohorts and evaluate the influence of factors such as symptom duration, comorbidities, and immunological status on oral viral dynamics.

In the Munich subgroup, all four patients showed a complete loss of viral RNA detection immediately after oral hygiene. In contrast, only 70% of patients in the Innsbruck cohort demonstrated this effect, suggesting interindividual variability. Such differences may be influenced by baseline viral burden, saliva composition, or adherence to the hygiene protocol. Notably, the methodology differed between sites: while Innsbruck used continuous Ct measurements, Munich relied on binary outcomes (positive/negative), which may partly explain the observed discrepancies.

Despite these limitations, the study provides important preliminary insights into the temporal variability of oral SARS-CoV-2 RNA levels and highlights the potential role of oral hygiene interventions and standardized saliva collection in diagnostic optimization. Larger, multicenter studies are needed to validate these findings and explore their implications for infection control and public health surveillance.

## 5. Conclusions

This study demonstrated that saliva-based SARS-CoV-2 detection via standardized mouth-rinse sampling shows high concordance with pharyngeal swab results and offers a practical, patient-friendly alternative for diagnostic use. The non-invasive nature and reduced exposure risk make it particularly suitable for settings requiring frequent testing or self-collection.

Observed Ct value dynamics revealed that oral viral load is subject to short-term fluctuations influenced by oral hygiene measures and behavioral factors such as food intake. While mechanical oral hygiene did not uniformly reduce viral detectability, meal ingestion appeared to cause a transient decline, followed by reaccumulation over time. Brief rinsing episodes also showed potential to temporarily lower detectable viral RNA levels.

### Future Prospects

These findings emphasize the importance of timing and pre-analytical standardization in saliva-based diagnostics. Standardized sampling conditions can improve the reliability and reproducibility of saliva testing. Manufacturers should provide clear instructions specifying the minimum time interval since the last meal or oral hygiene activity, or at least indicate that these factors can influence test results.

Future protocols should consider behavioral variables to enhance diagnostic reliability and reproducibility. Further research is needed to confirm these observations and to develop evidence-based guidelines for saliva-based sampling in infectious disease surveillance and outbreak preparedness.

## Data Availability

The data presented in this study are available on reasonable request from the corresponding author. The data are not publicly available due to institutional data protection and patient privacy regulations.
